# The Mediating Role of Psychological Capital in the Relationship Between Professional Nursing Governance, Nurses’ Perceived Care Quality and Job Satisfaction

**DOI:** 10.1155/jonm/3392843

**Published:** 2026-09-22

**Authors:** Melek Karatuzla, Betül Sönmez

**Affiliations:** ^1^ Department of Nursing, Faculty of Health Sciences, Istanbul Yeni Yuzyil University, Istanbul, Türkiye; ^2^ Department of Nursing Management, Florence Nightingale Faculty of Nursing, Istanbul University-Cerrahpaşa, Istanbul, Türkiye

**Keywords:** job satisfaction, nurses, nursing management, perceived care quality, professional nursing governance, psychological capital

## Abstract

**Background:**

Decision‐making structures are a fundamental component of the professional practice environments and are closely associated with nursing excellence.

**Aim:**

This study examined the relationships between professional nursing governance, nurses’ perceived care quality and job satisfaction, as well as the mediating role of psychological capital in these relationships.

**Method:**

This cross‐sectional, descriptive and correlational study was conducted with 514 nurses working in Istanbul between October 2024 and January 2025. Data were collected face to face and online using the Hess Professional Nursing Governance Index (IPNG) 3.0, the Psychological Capital Questionnaire (PCQ‐12) Short Form, the Nursing Care Quality Assessment Scale and the Nurse Satisfaction Questionnaire. Descriptive and correlation analyses were performed, and the hypothesised model was tested using structural equation modelling.

**Results:**

Professional nursing governance was positively related to psychological capital (standardised *β* = 0.453, *p* < 0.01), unit‐level perceived nursing care quality (standardised *β* = 0.311, *p* < 0.01), hospital‐level perceived nursing care quality (standardised *β* = 0.328, *p* < 0.01) and job satisfaction (standardised *β* = 0.376, *p* < 0.01). Psychological capital also demonstrated positive relationships with unit‐level perceived nursing care quality (standardised *β* = 0.339, *p* < 0.01), hospital‐level perceived nursing care quality (standardised *β* = 0.362, *p* < 0.01) and job satisfaction (standardised *β* = 0.300, *p* < 0.01).

**Conclusion:**

The findings suggest that higher levels of professional nursing governance are associated with stronger psychological capital, more favourable evaluations of care quality and greater job satisfaction among nurses. Psychological capital may represent a potential mechanism underlying the observed associations between professional nursing governance and these outcomes.

**Implication for Nursing Management:**

To enhance the effectiveness of nursing management, a practice environment integrated with administrative processes should be established, granting nurses genuine and explicit decision‐making authority. Managers may consider adopting a supportive and facilitative role rather than a supervisory approach, which may be associated with stronger psychological capital, greater professional autonomy, and higher organisational commitment and more favourable nurse outcomes, including higher perceived care quality and job satisfaction.

## 1. Introduction

Improving care quality and increasing nurse satisfaction in healthcare settings are closely related not only to organisational structures but also to how these structures are perceived and adopted by nurses [1]. According to Hess, professional governance, regarded as an innovation in nursing management, increases nurses’ influence in administrative areas while legitimising their control over professional practice [2]. For more than 40 years, healthcare organisations have sought to implement professional governance to enhance nurses’ professional autonomy and responsibility by enabling them to take an active role in organisational decision‐making [3]. Most previous studies have reported positive associations between professional nursing governance and employee empowerment, job satisfaction and perceived care quality [4, 5]. However, the fact that governance practices do not yield equally positive results for all nurses suggests that individual psychological resources may be associated with how nurses perceive and benefit from governance structures.

Psychological capital, which refers to an individual’s use of positive psychological attributes (hope, optimism, self‐efficacy, and resilience) to achieve goals and enhance performance [6], is considered a potential explanatory factor linking organisational factors with nurse outcomes, including nurses’ perceived care quality and job satisfaction. Differences in psychological resources may partly explain why governance structures do not produce equally positive outcomes for all nurses. Recent studies have shown that psychological capital is associated with desirable work‐related attitudes and behaviours, including job satisfaction, organisational commitment, organisational citizenship behaviour and professional performance [7, 8]. In empowering work environments, the role of psychological factors, alongside structural factors, can yield significant benefits, particularly for managers seeking to maximise the value of governance structures.

Despite growing interest in the outcomes of professional governance, significant gaps remain in the literature. Previous studies have examined organisational factors such as structural empowerment, autonomy, control over nursing practice and work environment characteristics in relation to job satisfaction and care quality [7, 8]. However, professional nursing governance is a distinct construct that focuses on the distribution of authority, accountability and decision‐making within professional nursing practice [2, 4]. Therefore, findings related to empowerment and other organisational characteristics cannot be assumed to fully explain the effects of professional nursing governance. Furthermore, although both organisational and individual factors have been associated with nurse outcomes, these factors have rarely been examined together within a single explanatory framework.

Psychological capital is regarded as an important individual resource that may be associated with care quality and job satisfaction through nurses’ ability to cope with stress, maintain work engagement and sustain positive attitudes towards their profession [9–11]. Although alternative explanations are possible, including that nurses with higher psychological capital may perceive governance structures more positively, previous research has frequently conceptualised psychological capital as a mediator between organisational factors and employee outcomes [9, 12, 13]. Nevertheless, the mediating role of psychological capital in the relationship between professional nursing governance and nurse outcomes has not been examined.

Like many healthcare systems, Türkiye faces challenges such as nursing workforce shortages, increasing patient care demands, retaining qualified nurses and maintaining high‐quality care [14]. Recent evidence from Türkiye also suggests that nurses’ professional autonomy and participation in decision‐making continue to be influenced by organisational and managerial factors, highlighting the importance of organisational structures that support nurses’ professional involvement and responsibility [15]. These challenges underscore the need for organisational structures that facilitate nurses’ participation and engagement in decision‐making processes. Although studies in Türkiye have examined empowerment, leadership, autonomy and job satisfaction among nurses, evidence on professional nursing governance remains limited. To our knowledge, no study in Türkiye has examined the relationships among professional nursing governance, psychological capital, nurses’ perceived care quality and job satisfaction within a single explanatory model. Therefore, this study addresses these gaps by examining the mediating role of psychological capital in the relationships among professional governance, job satisfaction and nurses’ perceived care quality, drawing on existing theoretical and empirical evidence.

## 2. Background

### 2.1. Professional Governance, Care Quality and Job Satisfaction

Professional governance is a multifaceted organisational characteristic that encompasses the structures and processes through which professionals direct, control and regulate their goal‐oriented efforts [16]. Although shared governance is often discussed within this framework, professional nursing governance is a broader concept that includes traditional, shared and self‐governance arrangements [17]. Within this continuum, shared governance represents an intermediate level in which authority and decision‐making responsibilities are shared between managers and staff nurses, whereas professional nursing governance more broadly concerns the distribution of authority, accountability, power and control over professional nursing practice [16].

Professional nursing governance can be considered theoretically through Kanter’s structural empowerment theory, which proposes that organisational structures influence employee attitudes and outcomes by providing access to information, resources, support and opportunities [17, 18]. Within professional governance structures, nurses are encouraged to participate in organisational decision‐making and exert greater influence over professional practice. As control over practice and resources shifts towards clinical nurses, they may experience increased professional involvement, autonomy and responsibility [17]. Consistent with this perspective, previous studies have reported positive associations between governance structures and nurse outcomes, including autonomy, nurse engagement, nurses’ perceived care quality and job satisfaction [18–23].

Despite growing evidence on the outcomes of professional nursing governance, less is known about the pathways underlying the observed associations between governance structures and nurse outcomes. Drawing on Kanter’s structural empowerment theory and conservation of resources (COR) theory, this study proposes that professional nursing governance is conceptualised as an organisational condition associated with greater participation, influence and control over professional practice [17, 19]. These governance experiences may be associated with stronger psychological resources, particularly psychological capital, which in turn may be associated with higher job satisfaction and nurses’ perceived care quality. Accordingly, this study will address the following hypothesis (H):

 H1: Professional nursing governance is positively associated with (a) psychological capital, (b) unit‐level perceived nursing care quality, (c) hospital‐level perceived nursing care quality and (d) nurse job satisfaction.

### 2.2. Psychological Capital as a Mediation Mechanism

Psychological capital is defined as an individual’s positive psychological state, characterised by hope, resilience, optimism, and self‐efficacy [24]. Within the framework of COR theory, psychological capital is viewed as a valuable personal resource that enables individuals to acquire, protect and maintain resources while effectively coping with workplace demands and stressors [25, 26]. From this perspective, organisational conditions may foster the development of psychological resources. Professional nursing governance provides nurses with opportunities to participate in decision‐making, exercise professional influence and exert greater control over their practice, which may be associated with higher levels of confidence, optimism, resilience and hope. Consequently, nurses working in environments characterised by higher levels of professional nursing governance may report higher levels of psychological capital.

Evidence supporting this mediating role has begun to emerge. Zhang et al. [13] reported that psychological capital significantly mediated the relationship between authentic leadership and nurses’ caring behaviours. Similarly, studies have suggested that psychological capital may represent an intermediary factor linking organisational factors with employee attitudes and outcomes [9, 27]. Therefore, psychological capital may help explain variability in the associations between professional nursing governance and nurse outcomes.

Alternative explanations are possible. For example, psychological capital may moderate rather than mediate the relationship between governance and outcomes, organisational climate may simultaneously influence both governance perceptions and psychological capital, or job satisfaction may influence perceptions of nursing care quality. However, the present study conceptualises psychological capital as a mediator because both structural empowerment theory and COR theory indicate that organisational conditions are proposed to shape employees’ psychological resources, which subsequently influence work attitudes and outcomes [17, 18, 25, 26].

Although previous studies have examined governance‐related outcomes and psychological capital separately, no study has simultaneously investigated professional nursing governance, psychological capital, nursing care quality and job satisfaction within a single explanatory model. Examining these relationships may lead to a better understanding of how governance structures are associated with organisational nurse‐reported patient and nurse outcomes. Accordingly, the following hypotheses were tested in this study.

 H2: Psychological capital is positively associated with (a) unit‐level perceived nursing care quality, (b) hospital‐level perceived nursing care quality and (c) nurse job satisfaction.
 H3: Psychological capital statistically mediates the relationships between professional nursing governance and (a) unit‐level perceived nursing care quality, (b) hospital‐level perceived nursing care quality and (c) nurse job satisfaction.

## 3. Material and Method

### 3.1. Participants

This cross‐sectional, descriptive and correlational study targeted nurses working in hospitals in Istanbul, Türkiye. As a comprehensive sampling frame of actively employed hospital nurses was not available, the minimum sample size was estimated using the formula for an unknown population. Based on a 95% confidence level and a 5% margin of error, a minimum sample of 384 nurses was required.

Nurses were recruited using a convenience sampling approach. The survey was distributed through professional nursing networks and online communication channels commonly used by nurses in Istanbul. Participation was voluntary, and nurses from various hospital settings were invited to take part.

The inclusion criteria were as follows: (1) voluntary participation in the study, (2) employment as a nurse in a hospital operating in Istanbul, (3) at least 6 months of work experience in the current institution and (4) completion of all study instruments. The exclusion criteria were as follows: (1) employment in healthcare settings other than hospitals and (2) not being actively employed during the data collection period.

### 3.2. Data Collection

The research data were collected using the “Nurse Demographic Information Form,” “Hess Professional Nursing Governance Index (IPNG) 3.0,” “Psychological Capital Scale Short Form (PCQ‐12),” “Nursing Care Quality Assessment Scale” and the “Nurse Job Satisfaction Questionnaire.” Data were collected from October 2024 to January 2025 using both online and paper‐based survey formats to facilitate participation. An online Google Forms survey containing the informed consent form and study instruments was distributed through professional nursing networks and communication channels. In addition, printed questionnaires were provided to nurses who preferred not to complete them online. The same instruments and procedures were used for both modes of data collection.

### 3.3. Data Collection Tools

Nurse Identification Information Form: The form developed by the researchers consists of 15 questions regarding the nurses’ personal (age, gender and education level), professional and institutional characteristics (city of employment, type of hospital, unit of employment, role, total length of service as a nurse, total length of service in the institution and unit, employment type, presence of a Ministry of Health–approved certificate, type and year of receipt of the certificate if applicable and the number of beds and nurses in the unit).

Hess Professional Nursing Governance Index (IPNG) 3.0: This index is developed by Hess to determine the professional governance levels of nurses and adapted into Turkish by Karatuzla [2, 28]. The original index comprised 50 items across six subdimensions: controls (8 items), influences (8 items), official authority (12 items), attending (8 items), access to information (9 items) and ability (5 items) [2]. The index is completed using a 5‐point Likert scale (1: *nursing management only*, 2: *primarily nursing management/administration with some staff nurse input*, 3: *equally shared by staff nurses and nursing management/administration*, 4: *primarily staff nurses with some nursing management/administration input* and 5: *just nurses*). In the Turkish adaptation, the original structure was preserved, but the number of items was reduced to 30 due to differences in nurses’ roles and practices in the Turkish healthcare system. Participants select one response per item, scored from 1 to 5, and the numerical value for each item is multiplied by the total number of IPNG items [28]. The original index yields a total score between 50 and 250, while the Turkish version ranges from 30 to 150. In the original index, scores between 201 and 250, and in the Turkish version between 121 and 150, indicate a self‐governing organisation with minimal administrative input, representing the optimal form of governance. The Cronbach’s alpha coefficient for the original index ranges from 0.82 to 0.90 for the subdimensions and 0.95 for the total index [2], while the Turkish version ranges from 0.75 to 0.93 for the subdimensions and 0.92 for the total index [28]. This study demonstrated high internal consistency, with subdimensions ranging from 0.87 to 0.94 and a total index of 0.96.

The Turkish adaptation of the IPNG followed established cross‐cultural adaptation procedures, including forward and backward translation, expert review, content validity assessment, pilot testing, exploratory factor analysis (EFA), confirmatory factor analysis (CFA) and reliability testing. In this study, conducted with a different sample from that used in the adaptation phase, the psychometric properties of the Turkish IPNG were re‐examined through CFA, convergent and discriminant validity analyses, and internal consistency assessment prior to testing the structural model. These additional analyses provided further evidence supporting the construct validity and reliability of the Turkish version in the current sample. Although the Turkish version contains fewer items than the original instrument, it retains the original six‐factor structure, preserving the conceptual domains of professional nursing governance while reflecting contextual differences in nursing roles and practices within the Turkish healthcare system.

Psychological Capital Short Form (PCQ‐12): The psychological capital scale, originally developed as a 24‐item scale by Luthans et al. (2007) to measure individuals’ psychological capital levels, was later shortened to a 12‐item version by Avey et al. [29, 30]. The Turkish validity and reliability study was conducted by Oruç (2018) [31]. The scale consists of 12 items across four subdimensions: self‐efficacy (three items), hope (four items), optimism (two items) and resilience (three items). The scale uses a 6‐point Likert format (1: *strongly disagree* and 6: *strongly agree*) to prevent participants from choosing the midpoint. High scores indicate high psychological capital [30, 31]. The Cronbach’s alpha coefficient for the original scale is reported as greater than 0.70 [30], while it was calculated as 0.931 for the Turkish version [31]. In this study, the scale showed high reliability with a Cronbach’s alpha coefficient of 0.942.

Assessment of Nursing Care Quality: The Nursing Care Quality Assessment Scale, developed by Aiken et al. and adapted into Turkish by İspir Demir et al., comprises four questions [32, 33]. In this study, two items were used to assess nurses’ overall perceptions of the quality of nursing care provided in their unit and the overall quality of patient care within their institution over the past year. These items capture nurses’ overall evaluations of care quality at the unit and organisational levels rather than specific dimensions of nursing care quality (e.g., technical care processes, communication or safety indicators) and are, therefore, intended as global outcome indicators. Responses to question 1 are rated on a 4‐point Likert scale (1: *poor*, 2: *average*, 3: *good* and 4: *excellent*), while responses to question 2 are rated on a 3‐point Likert scale (1: *worsened*, 2: *remained the same* and 3: *improved*). The reliability of the instrument was evaluated using test–retest analysis, with ICC values ranging from 0.70 to 0.79, indicating good to excellent reliability [33].

Nurse Job Satisfaction Global Question: The question “How satisfied are you with your job?” was evaluated using the method developed by Aiken et al. and adapted into Turkish by İspir Demir et al. [32, 33]. Responses are rated on a 4‐point Likert scale (1: *very dissatisfied* and 4: *very satisfied*). In the Turkish adaptation study, responses may be categorised as dissatisfied (1‐2) and satisfied (3‐4) for descriptive purposes [33]. However, in the present study, the original four‐point response format was retained and analysed as a continuous observed variable in all statistical analyses. Although this measure does not assess specific dimensions of job satisfaction, it provides a global indicator of overall job satisfaction and demonstrated acceptable test–retest reliability in the Turkish validation study (ICC = 0.84) [33].

### 3.4. Data Analysis

Data from the study were analysed using IBM SPSS Statistics 26.0 (IBM Corp., Armonk, NY, USA) and IBM SPSS AMOS 24 (Scientific Software International, Skokie, IL, USA). The final sample comprised 514 nurses. This sample size exceeded commonly recommended minimums for structural equation modelling and was considered adequate for the complexity of the proposed model, supporting stable parameter estimation and hypothesis testing [34]. A total of 554 questionnaires were collected. Of these, 40 were excluded because of incomplete responses, resulting in 514 complete questionnaires included in the final analysis. No item‐level imputation was performed, and only complete cases were included in the analyses.

Descriptive statistics were presented as means and standard deviations, or medians [minimum–maximum] for continuous variables, and as frequencies and percentages for categorical variables. The normality assumption was evaluated using skewness and kurtosis coefficients. The skewness and kurtosis coefficients of the scores obtained from the scales and their subdimensions were within the range of −1.5 to +1.5, indicating that these scores had a normal distribution [35]. In addition, several study variables were assessed using single ordinal items: unit‐level and hospital‐level perceived nursing care quality (4‐point and 3‐point scales, respectively) and job satisfaction (4‐point scale). Rather than modelling these as latent constructs, we entered them into the structural equation model as observed continuous outcomes. Skewness and kurtosis coefficients were examined and fell within acceptable limits for normal approximation (skewness ranged from −0.41 to −0.01, and kurtosis ranged from −1.09 to −0.73) [36]. This treatment of single‐item ordinal indicators as continuous is a recognised approach for global, unidimensional evaluations of this kind particularly where the underlying distribution approximates normality [36–39]. However, the relatively small number of response categories may still impose some estimation constraints when these ordinal outcomes are treated as continuous [38]. This analytical choice is, therefore, acknowledged as a methodological limitation.

The correlation between the scales was examined using the Pearson correlation coefficient. In the analysis of the formulated hypothesis model, the relationships between the observed and latent variables were estimated using SEM with IBM SPSS AMOS 24. The bootstrapping method (5000 bootstrap samples) was used to estimate the indirect effects specified in the mediation model. The statistical significance level was set at *p* < 0.01. Structural equation modelling was used to test the theoretically proposed relationships among the study variables and to evaluate the indirect effects specified in the mediation model. Given the cross‐sectional nature of the data, the proposed mediation model should be interpreted as a theoretically informed explanatory framework rather than as evidence of temporal or causal relationships. Therefore, the mediation analyses do not establish causality but assess whether the observed data are consistent with the hypothesised relationships.

Demographic and organisational characteristics were not included as control variables in the structural model, as the primary aim of the study was to test the theoretically specified relationships among professional governance, psychological capital, nurses’ perceived care quality and job satisfaction.

Nurses’ perceived care quality and job satisfaction were measured using single‐item indicators. Such global measures are widely used in nursing workforce research to assess overall evaluations of care quality and job satisfaction. Because these variables represented global evaluations rather than multidimensional constructs, they were included in the structural equation model as single‐indicator outcome variables. Their inclusion was based on their theoretical importance in the proposed model.

### 3.5. Ethical Considerations

The study was submitted to the Beykent University Scientific Research and Publication Ethics Committee for Social and Human Sciences for ethical approval, which was granted (date: 28.03.2022; revised date: 16.02.2023; number: 93,634). The approval covered the entire study protocol, including both online and face‐to‐face data collection procedures, and remained valid throughout the data collection period (October 2024–January 2025).

The data collection tools in Google Forms included an informed consent form outlining the purpose, duration and procedures of the research. In the online informed consent form, participants ticked a box instead of signing. Participants were informed that they needed to tick the box to proceed with the research. Before printed data were collected, the informed consent form was presented to participants for signature.

All data were collected anonymously, and no identifying personal information was recorded. Online survey data were collected via Google Forms with restricted access, and only the researchers had access to the dataset. All electronic data were stored in password‐protected files on secure devices accessible only to the research team. Printed questionnaires were kept in locked storage. Data were used solely for scientific purposes and handled in accordance with confidentiality principles.

All procedures were conducted in accordance with the ethical standards of institutional and national investigative committees and the principles of the 1964 Helsinki Declaration, as revised in 2000.

## 4. Results

### 4.1. Characteristics of Nurses

The participants’ personal and professional characteristics are presented in Table 1. The mean age of the nurses in the study (*n* = 514) was 29.28 years (SD = 6.25), with ages ranging from 21 to 51. Of the nurses, 83.3% (*n* = 428) were female, and 66.3% (*n* = 341) held a bachelor’s degree. Regarding work characteristics, 37.4% (*n* = 192) worked in wards, 84.4% (*n* = 434) were staff nurses, and 70.6% (*n* = 363) worked shifts. It was found that 84.4% (*n* = 432) of the nurses did not have a Ministry of Health–approved certificate specific to their unit. The average total year of professional experience was 6.92 (SD = 6.54; minimum 1 and maximum 32) (Table 1).

**TABLE 1:** Characteristics of nurses (*N* = 514).

Variables	Categories	Frequency (*N*)	Percentage (%)	Mean ± SD	Median (min–max)
Age	≤ 25 years	147	28.6	29.28 ± 6.25	27.00 (21–51)
	26–30 years	217	42.2		
	≥ 31 years	150	29.2		

Sex	Female	428	83.3		
	Male	86	16.7		

Education level	Vocational high school (health services)	53	10.3		
	Associate degree	55	10.7		
	Bachelor’s degree	341	66.3		
	Postgraduate degree	65	12.6		

Hospital where you work	Public hospital	38	7.4		
	Training and research hospital	282	54.9		
	City hospital	26	5.1		
	Foundation university hospital	11	2.1		
	Private hospital	157	30.5		

Unit worked in	Inpatient unit (ward)	192	37.4		
	Outpatient clinic	21	4.1		
	Emergency department	95	18.5		
	Intensive care unit	104	20.2		
	Specialised unit (e.g., interventional unit, dialysis)	36	7		
	Operating room	39	7.6		
	Other	27	5.3		

Role	Staff nurse	434	84.4		
	Specialist nurse	19	3.7		
	Charge nurse	47	9.1		
	Other^∗∗^	17	2.8		

Work pattern	Shift work	363	70.6		
	Permanent day shift	151	29.4		

Presence of Ministry of Health–Approved Certification	Yes	82	16		
	No	432	84		

Total years of professional experience	1–5 year	279	54.3	6.92 ± 6.54	5.00 (1–32)
	6–10 year	131	25.5		
	11–15 year	45	8.8		
	≥ 16 years	59	11.5		

Years of experience in the current institution	1–5 years	353	68.7	4.98 ± 4.60	3.00 (1–32)
	6–10 years	96	18.7		
	11–15 years	33	6.4		
	≥ 16 years	32	6.2		

Years of experience in the current unit	1–5 years	409	79.6	3.78 ± 3.65	2.00 (1–30)
	6–10 years	68	13.2		
	11–15 years	17	3.3		
	≥ 16 years	20	3.9		

Number of beds in the unit	≤ 20 beds	275	53.5	21.21 + 15.75	18.00 (< 1–50)
	> 20 beds	239	46.5		

Number of nurses in the unit	≤ 25 nurses	348	67.7	24.94 + 24.61	12.00 (< 1–105)
	**>** 25 nurses	166	32.3		

*Note:* Min: minimum, Max: Maximum.

Abbreviation: SD, standard deviation.

^∗∗^Director of health care services/nursing services director/deputy director/coordinator.

Although the sample mainly comprised female nurses, bachelor’s degree holders, staff nurses and shift workers, this distribution reflects the typical workforce composition in hospital settings in Istanbul and should be considered when interpreting the external validity of the findings.

Demographic and professional characteristics were examined descriptively and were not included as grouping or control variables in the structural model, which focused on testing the hypothesised relationships among study constructs.

### 4.2. Initial Analyses

The study’s measurement model includes four variables: professional governance (30 items), psychological capital (12 items), nursing care quality (2 questions) and nurse job satisfaction (1 question). As care quality and nurse job satisfaction were measured separately, CFA was conducted only on the professional governance and psychological capital scales.

The CFA models were specified according to the original theoretical factor structures of the instruments, and no post hoc model modifications or correlated error terms were introduced. The standardised factor loadings for the observed variables in the CFA were 0.55–0.94 for professional governance and 0.77–0.95 for psychological capital. These results indicate that the scales showed statistically significant standardised factor loadings.

Model fit indicated an overall acceptable fit for the measurement model. For professional governance, fit indices were *χ* ^2^/sd = 4.334, RMR = 0.064, GFI = 0.913, AGFI = 0.910, NFI = 0.954, IFI = 0.951, TLI = 0.960, CFI = 0.952 and RMSEA = 0.081. For psychological capital, fit indices were *χ* ^2^/sd = 3.798, RMR = 0.066, GFI = 0.943, AGFI = 0.903, NFI = 0.972, IFI = 0.979, TLI = 0.970, CFI = 0.979 and RMSEA = 0.074. While most indices indicated acceptable model fit, the RMSEA value for the professional governance scale (0.081) falls within the borderline range, suggesting a reasonable but not excellent model fit.

To assess the potential impact of common method variance, Harman’s single‐factor test was performed. All measurement items were entered into an unrotated EFA. The first factor accounted for 38.27% of the total variance, which was below the recommended threshold of 50%, suggesting that common method variance is unlikely to pose a serious threat to the validity of the study findings [40].

For the professional governance and psychological capital scales, the results for convergent and discriminant validity showed that the composite reliability (CR) values exceeded 0.70, the mean average variance extracted (AVE) values were above 0.50, and all standardised factor loadings (*λ*) were greater than 0.50, supporting convergent validity. Furthermore, AVE values were greater than both the maximum shared squared variance (MSV) and the average shared variance (ASV), supporting discriminant validity [34, 41] (Table 2).

**TABLE 2:** Convergent and discriminant validities of the Hess index of professional nursing governance and the psychological capital short form (*N* = 514).

	**CR**	**AVE**	**MSV**	**ASV**	**1**	**2**	**3**	**4**	**5**	**6**

*IPNG*										
1	0.918	0.614	0.464	0.365	0.784					
2	0.940	0.798	0.613	0.354	0.681	0.893				
3	0.868	0.686	0.257	0.165	0.507	0.320	0.828			
4	0.950	0.707	0.613	0.381	0.666	0.783	0.373	0.841		
5	0.879	0.648	0.410	0.338	0.583	0.604	0.438	0.640	0.805	
6	0.909	0.714	0.383	0.273	0.568	0.474	0.367	0.549	0.619	0.845

	**CR**	**AVE**	**MSV**	**ASV**	**1**	**2**	**3**	**4**		

*PCQ-12*										
1	0.904	0.759	0.507	0.420	0.871					
2	0.939	0.793	0.602	0.488	0.712	0.891				
3	0.918	0.790	0.461	0.368	0.538	0.595	0.889			
4	0.910	0.835	0.602	0.509	0.680	0.776	0.679	0.914		

*Note:* ASV: average shared square variance; IPNG: Hess Index of Professional Nursing Governance; 1. controls, 2. influences, 3. offıcıal authorıty, 4. attendıng, 5. access to informatıon, and 6. abılıty; PCQ‐12: Psychologıcal Capıtal Short Form; 1. self‐efficacy, 2. hope, 3. resilience, and 4. optimism.

Abbreviations: AVE, average variance extracted; CR, composite reliability; MSV, maximum squared variance.

Cronbach’s alpha coefficients were 0.96 for professional governance and 0.94 for psychological capital, indicating excellent internal consistency.

### 4.3. Descriptive Findings and Correlation Analyses Regarding the Variables

Detailed results for the mean scores of the variables and Pearson correlation analyses are presented in Table 3. The mean total score for IPNG was 66.43 ± 22.78, and the mean total score for PCQ‐12 was 4.09 ± 1.24. The mean unit‐level score for nurses’ perceived nursing care quality was 2.71 ± 0.95; the mean hospital‐level score for nurses’ perceived nursing care quality was 2.27 ± 0.67; and the mean nurse job satisfaction score was 2.58 ± 0.90.

**TABLE 3:** Descriptive findings and correlation matrix for the variables (*N* = 514).

	1	2	3	4	5	6	7	8	9	10	11	12	13	14	15
1	(0.958)														
2	0.823^∗∗^	(0.917)													
3	0.785^∗∗^	0.635^∗∗^	(0.939)												
4	0.645^∗∗^	0.462^∗∗^	0.295^∗∗^	(0.867)											
5	0.802^∗∗^	0.611^∗∗^	0.726^∗∗^	0.336^∗∗^	(0.934)										
6	0.796^∗∗^	0.559^∗∗^	0.527^∗∗^	0.440^∗∗^	0.553^∗∗^	(0.895)									
7	0.729^∗∗^	0.521^∗∗^	0.447^∗∗^	0.335^∗∗^	0.490^∗∗^	0.558^∗∗^	(0.908)								
8	0.459^∗∗^	0.415^∗∗^	0.274^∗∗^	0.349^∗∗^	0.309^∗∗^	0.371^∗∗^	0.374^∗∗^	(0.942)							
9	0.352^∗∗^	0.282^∗∗^	0.212^∗∗^	0.253^∗∗^	0.237^∗∗^	0.303^∗∗^	0.318^∗∗^	0.816^∗∗^	(0.927)						
10	0.374^∗∗^	0.368^∗∗^	0.192^∗∗^	0.326^∗∗^	0.237^∗∗^	0.284^∗∗^	0.294^∗∗^	0.862^∗∗^	0.632^∗∗^	(0.943)					
11	0.378^∗∗^	0.346^∗∗^	0.229^∗∗^	0.280^∗∗^	0.259^∗∗^	0.299^∗∗^	0.313^∗∗^	0.799^∗∗^	0.474^∗∗^	0.560^∗∗^	(0.913)				
12	0.439^∗∗^	0.403^∗∗^	0.287^∗∗^	0.317^∗∗^	0.308^∗∗^	0.360^∗∗^	0.332^∗∗^	0.887^∗∗^	0.628^∗∗^	0.730^∗∗^	0.633^∗∗^	(0.908)			
13	0.464^∗∗^	0.425^∗∗^	0.267^∗∗^	0.368^∗∗^	0.332^∗∗^	0.399^∗∗^	0.323^∗∗^	0.480^∗∗^	0.396^∗∗^	0.434^∗∗^	0.348^∗∗^	0.441^∗∗^			
14	0.492^∗∗^	0.428^∗∗^	0.333^∗∗^	0.413^∗∗^	0.352^∗∗^	0.386^∗∗^	0.326^∗∗^	0.513^∗∗^	0.394^∗∗^	0.451^∗∗^	0.404^∗∗^	0.478^∗∗^	0.578^∗∗^		
15	0.512^∗∗^	0.472^∗∗^	0.319^∗∗^	0.359^∗∗^	0.412^∗∗^	0.418^∗∗^	0.362^∗∗^	0.480^∗∗^	0.402^∗∗^	0.380^∗∗^	0.352^∗∗^	0.483^∗∗^	0.478^∗∗^	0.480^∗∗^	
Mean (SD)	66.43 (22.78)	16.70 (6.58)	7.55 (3.92)	7.91 (3.32)	15.14 (7.35)	9.34 (4.00)	9.77 (3.91)	4.09 (1.24)	4.19 (1.54)	4.21 (1.39)	3.88 (1.55)	4.09 (1.45)	2.71 (0.95)	2.27 (0.67)	2.58 (0.90)
Min‐Max	30–150	7–35	4–20	3–15	8–40	4–20	4–20	1–6	1–6	1–6	1–6	1–6	1–4	1–3	1–4

*Note:* Pearson correlation analysis, 1. IPNG: Hess Index of Professional Nursing Governance; 2. IPNG: controls; 3. IPNG: influences; 4. IPNG: offıcial authorıty; 5. IPNG: attending; 6. IPNG: access to information; 7. IPNG: ability; 8. PCQ‐12: Psychologıcal Capıtal Short Form; 9. PCQ‐12: self‐efficacy; 10. PCQ‐12: hope; 11. PCQ‐12: resilience; 12. PCQ‐12: optimism; 13. nursing care qualıty‐unıt; 14. nursing care qualıty‐hospıtal; 15. job satisfaction.

^∗∗^ *p* < 0.01.

A positive, statistically significant correlation was found between professional nursing governance and psychological capital (*r* = 0.459, *p* < 0.01). Positive and significant correlations were found between professional nursing governance and unit‐level perceived nursing care quality (*r* = 0.464, *p* < 0.01), hospital‐level perceived nursing care quality (*r* = 0.492, *p* < 0.01) and nurse job satisfaction (*r* = 0.512, *p* < 0.01). Positive and significant correlations were also found between psychological capital and unit‐level perceived nursing care quality (*r* = 0.480, *p* < 0.01), hospital‐level perceived nursing care quality (*r* = 0.513, *p* < 0.01) and nurse job satisfaction (*r* = 0.480, *p* < 0.01) (Table 3).

Although these correlations are statistically significant, their magnitudes indicate moderate association sizes. Given the large sample size (*n* = 514), statistical significance should be interpreted with caution, and emphasis should be placed on association sizes rather than *p* values alone.

As all variables were based on self‐reported perceptions, the observed associations reflect perceived rather than objective organisational and clinical outcomes. Therefore, the findings should be interpreted as relationships among subjective evaluations rather than as direct indicators of organisational performance or objective care quality.

### 4.4. Findings on the Research Hypothesis Model

**FIGURE 1:**
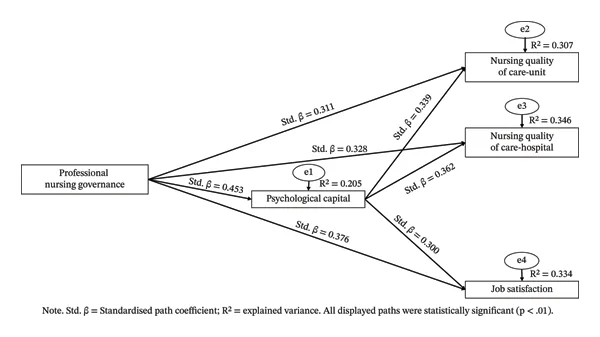
Structural equation model of the hypothesised relationships.

The hypothesised model of the relationships among professional nursing governance, psychological capital, unit‐level perceived nursing care quality, hospital‐level perceived nursing care quality and nurse job satisfaction, as well as the mediating role of psychological capital in these relationships, was examined using SEM within a theoretically specified explanatory framework. Given the cross‐sectional design, the model should be interpreted as explanatory rather than a confirmatory test of causality. The path analysis for the hypothesised model is presented in Figure 1.

SEM fit indices are presented in Table 4. The chi‐square statistic was relatively small (*χ* ^2^ = 3.236, df = 2), and the normalised chi‐square (*χ* ^2^/df = 1.618) indicated an acceptable model fit [34, 35]. Error‐based fit indices, RMSEA (0.051) and RMR (0.042) also suggested an acceptable model fit [42]. Furthermore, incremental fit indices, NFI = 0.954, IFI = 0.956, TLI = 0.989 and CFI = 0.965, all exceeded the recommended criterion of 0.95, indicating a good model fit [42, 43]. The absolute fit indices, GFI = 0.985 and AGFI = 0.971, also exceeded the widely accepted threshold of 0.90, supporting the adequacy of the model [42]. Overall, these findings indicate that the proposed structural model provides an acceptable to good fit to the observed data.

**TABLE 4:** Goodness‐of‐fit values for structural equation models (*N* = 514).

Criteria	*χ* ^2^/df	RMR	GFI	AGFI	NFI	IFI	TLI	CFI	RMSEA
Good fit	≤ 3	≤ 0.05	≥ 0.95	≥ 0.95	≥ 0.95	≥ 0.95	≥ 0.95	≥ 0.95	≤ 0.05
Acceptable	≤ 5	≤ 0.08	≥ 0.90	≥ 0.90	≥ 0.90	≥ 0.90	≥ 0.90	≥ 0.90	≤ 0.08
Model fit values	1618	0.042	0.971	0.985	0.954	0.956	0.989	0.965	0.051

*Note:* *χ* ^2^/df, corrected chi‐square; RMR, root mean square residual.

Abbreviations: AGFI, adjusted goodness of fit index; CFI, comparative fit index; GFI, goodness of fit index; IFI, incremental fit index; NFI, normed fit index; RMSEA, root mean square error of approximation; TLI, Tucker–Lewis index.

Path coefficients were estimated using SEM to examine the hypothesised relationships among the study variables. Professional nursing governance was positively associated with psychological capital (standardised *β* = 0.453, *p* < 0.01), unit‐level perceived nursing care quality (standardised *β* = 0.311, *p* < 0.01), hospital‐level perceived nursing care quality (standardised *β* = 0.328, *p* < 0.01) and nurse job satisfaction (standardised *β* = 0.376, *p* < 0.01). Based on these results, H1 was fully supported. Furthermore, psychological capital was also positively associated with unit‐level perceived nursing care quality (standardised *β* = 0.339, *p* < 0.01), hospital‐level perceived nursing care quality (standardised *β* = 0.362, *p* < 0.01) and nurse job satisfaction (standardised *β* = 0.300, *p* < 0.01) (Table 5). Thus, H2 was fully supported.

**TABLE 5:** Analysis results regarding the hypothesis model (*N* = 514).

Variables	*β*_1_	*β*_0_	S.E.	C.R.	*p*	*R* ^2^	%95 CI		*p*
							LB	UB	
*Direct effect*									
IPNG ⟶ PCQ‐12	0.453	0.750	0.06	11,497	0.000	0.205	0.378	0.511	0.01
PCQ‐12 ⟶ NCQU	0.339	0.260	0.03	8217	0.000	0.307	0.272	0.412	0.01
IPNG ⟶ NCQU	0.311	0.394	0.05	7536	0.000		0.237	0.380	0.01
PCQ‐12 ⟶ NCQH	0.362	0.199	0.02	9037	0.000	0.346	0.288	0.424	0.01
IPNG ⟶ NCQH	0.328	0.298	0.03	8188	0.000		0.269	0.396	0.01
PCQ‐12 ⟶ JS	0.300	0.219	0.03	7427	0.000	0.334	0.233	0.358	0.01
IPNG ⟶ JS	0.376	0.454	0.04	9292	0.000		0.314	0.437	0.01

*Indirect effect*									
IPNG ⟶ NCQU	0.153	0.195	0.02	7222			0.112	0.202	0.01
IPNG ⟶ NCQH	0.164	0.149	0.02	5730			0.115	0.205	0.01
IPNG ⟶ JS	0.136	0.164	0.02	7454			0.098	0.171	0.01

*Total effect*									
IPNG ⟶ NCQU	0.434	0.589	0.04	12,020			0.399	0.524	0.01
IPNG ⟶ NCQH	0.492	0.447	0.03	12,081			0.435	0.551	0.01
IPNG ⟶ JS	0.512	0.619	0.04	13,456			0.453	0.568	0.01

*Note: p* < 0.01, *R* ^2^: explained variance; *β*_1_: standardised coefficients; *β*_0_: unstandardised coefficients; IPNG: Hess index of professional nursing governance; PCQ‐12: psychologıcal capıtal short form.

Abbreviations: CI, confidence interval; C.R, critical ratio; JS, job satisfaction; LB, lower bound; NCQH, nursing care qualıty‐hospıtal; NCQU, nursing care qualıty‐unıt; S.E, standard error; UB, upper bound.

All reported path coefficients are standardised estimates obtained from the structural model. These coefficients should not be interpreted as equivalent to bivariate correlations as SEM estimates relationships after accounting for measurement error and the simultaneous associations of other variables in the model. Consequently, the standardised path coefficient between professional nursing governance and psychological capital may be larger than the corresponding zero‐order correlation.

The mediating role of psychological capital in the associations between professional nursing governance, unit‐level perceived nursing care quality, hospital‐level perceived nursing care quality and nurse job satisfaction was examined using the bootstrap method (5000 samples). Professional nursing governance was directly and positively significantly associated with unit‐level perceived nursing care quality (std. *β* = 0.311, *p* < 0.01), hospital‐level perceived nursing care quality (std. *β* = 0.328, *p* < 0.01) and nurse job satisfaction (std. *β* = 0.376, *p* < 0.01). Furthermore, professional nursing governance showed significant indirect associations with unit‐level perceived nursing care quality (std. *β* = 0.153, 95% [CI 0.112, 0.202]), hospital‐level perceived nursing care quality (std. *β* = 0.164, 95% [CI 0.115, 0.205]) and nurse job satisfaction (std. *β* = 0.136, 95% [CI 0.098, 0.171]) through psychological capital. The total standardised associations, representing the combined direct and indirect associations, were *β* = 0.464 for unit‐level perceived nursing care quality, *β* = 0.492 for hospital‐level perceived nursing care quality and *β* = 0.512 for nurse job satisfaction (Table 5). These findings are consistent with a partial mediating role of psychological capital in the associations between professional nursing governance and unit‐level perceived nursing care quality, hospital‐level perceived nursing care quality and nurse job satisfaction. Based on these results, H3 was supported.

The *R* ^2^ values indicate that the model explains a moderate proportion of variance in the outcome variables; however, a significant amount of unexplained variance remains, suggesting that additional organisational and individual factors may also be associated with nurses’ perceived care quality and job satisfaction.

## 5. Discussion

Professional nursing governance is a decentralised, modern management approach that supports nurses’ authority, autonomy and accountability in decision‐making processes while fostering a collaborative environment. This management model enables nurses to participate actively in organisational governance structures and has been associated with higher job satisfaction and higher nurses’ perceived care quality in previous studies [44]. In this context, the study examined the relationships among professional nursing governance, nurses’ perceived care quality and nurse job satisfaction, with a focus on the mediating role of psychological capital. The findings indicated that professional nursing governance was significantly associated with nurses’ perceived care quality at both unit and hospital levels, as well as with nurse job satisfaction, directly and indirectly through psychological capital within the tested model.

Self‐efficacy, optimism, hope and resilience essential characteristics of psychological capital tend to be higher in environments characterised by supportive and empowering nursing management [45, 46]. This study found a significant positive relationship between professional nursing governance and psychological capital. No studies directly examining this relationship were available. However, previous studies have shown a positive and significant relationship between structural empowerment, a fundamental element of professional governance and psychological capital [7, 41]. In this study, professional nursing governance was associated with higher levels of nurses’ psychological capital (self‐efficacy, hope, resilience and optimism), which may represent one potential explanatory pathway underlying the observed associations, a finding that is consistent with Kanter’s structural empowerment theory. Clavelle et al. state that professional governance provides a structural framework that empowers nurses to fully use their expertise, knowledge and wisdom within the health system [47]. Systemic barriers, such as bureaucratic decision‐making processes and perceived power imbalances between nurses and physicians, restrict nurses’ ability to act independently [48]. Many managers still maintain a traditional management approach, where management retains significant control over decision‐making, thereby undermining genuine professional management practices [49]. In this study, results at the level of professional governance, which prioritised nursing management and considered the contributions of some nurses, showed that there are areas in nursing where professional governance requires improvement. In particular, the relatively lower scores observed in governance‐related dimensions suggest that nurses may perceive limited influence on strategic and administrative decisions. This finding may indicate that opportunities for participation in higher level organisational processes remain restricted despite the presence of governance structures. Such a pattern may partly explain why the observed associations, although significant, were not stronger. The subdimension with the lowest score indicates that nurses have limited influence at higher levels on issues such as budgets, personal rights, salaries, raises, benefits and the determination of new clinical and administrative positions. The findings are also consistent with Hobfoll’s theory of resource conservation [50]. These resources are linked to lower burnout and increased performance capacity. Greater collaborative governance may be associated with nurses feeling more empowered and perceiving increased opportunities to contribute to decision‐making processes in their field [51]. Although the observed association was statistically significant, the magnitude of the relationship suggests that professional governance is only one of several organisational factors associated with nurses’ psychological capital. Other workplace characteristics, such as leadership style, team climate and organisational support, may also contribute to the development of psychological resources.

Furthermore, autonomy may be associated not only with individual nurse outcomes but also with nurses’ perceived care quality. Professional governance is a work model in which nurses and interdisciplinary teams follow an organised decision‐making process regarding practice standards, quality improvement, professional development and research [21]. This study also found a significant positive association between professional nursing governance and nurses’ perceived quality at both unit and hospital levels. However, it should be noted that care quality in the present study was assessed through nurses’ subjective evaluations rather than objective quality indicators, patient outcomes, adverse events or patient‐reported measures. Therefore, these findings should be interpreted as reflecting nurses’ perceived care quality rather than direct evidence of improved patient outcomes. A study conducted in California, New Jersey, Pennsylvania and Florida found that hospitals where nurses participated in organisational decision‐making had 44% higher patient care quality [20]. Similarly, previous studies have reported that nurses’ perceptions of professional governance are associated with care culture and nursing‐sensitive indicators [52, 53]. National and international studies examining the positive impact of structural strengthening on patient care quality also support these findings [33, 54, 55].

Another finding of the study is a significant positive relationship between professional nursing governance and nurse job satisfaction. Previous studies have also reported a significant positive relationship between professional nursing governance and nurse job satisfaction [17, 18, 23, 56]. A supportive environment in which nurses actively participate in setting organisational standards and policies, and in which their opinions are valued, is associated with higher job satisfaction [57] and with nurses’ sense of responsibility [58]. A randomised controlled trial conducted in Saudi Arabia [17] found that shared governance practices, such as establishing unit‐based councils, positively influenced nurses’ perceptions of professional governance and increased their commitment and job satisfaction. Another organisational characteristic associated with organisational job satisfaction is the development of a work model based on professional governance, interprofessional collaboration and mutual respect among healthcare team members. Studies have reported that collaborative practices are associated with improved team dynamics, communication, organisational commitment and job satisfaction [59]. This study further supports the existence of a positive association among Turkish nurses. The relatively stronger association observed between professional nursing governance and job satisfaction, compared with some other outcomes in the model, may reflect that governance structures are experienced directly by nurses in their daily work. Opportunities to participate in decision‐making processes, express professional opinions, and influence practice‐related issues may be more immediately reflected in nurses’ job satisfaction than in broader evaluations of care quality.

The hypothesised model also revealed a significant positive relationship between psychological capital and nurses’ perceived care quality, both within the unit and across the hospital. Previous studies have reported positive associations between psychological capital, employee performance and engagement. The relevant literature is consistent with these findings (e.g., [13, 45]). One possible explanation is that higher levels of self‐efficacy and resilience may help nurses cope more effectively with workplace challenges and are associated with better performance and higher perceived care quality [60, 61]. Higher levels of psychological capital are also associated with lower work‐related stress and higher job satisfaction [62]. Similarly, this study showed a significant positive correlation between psychological capital and nurse job satisfaction. Research involving nurses supports these findings [13, 63]. Zhang et al. found that the relationship between psychological capital and job satisfaction was stronger during the COVID‐19 pandemic, especially among specialist nurses [13]. This finding may be explained by the challenges introduced during the COVID‐19 pandemic, which challenged existing roles, changed organisational priorities and altered staff responsibilities.

These findings are consistent with the hypothesis that psychological capital may partially mediate the relationship between professional nursing governance and nurses’ perceived care quality and job satisfaction. The indirect associations observed in the model indicate that governance may be associated with perceptions of care quality and job satisfaction partly through nurses’ psychological resources. However, the magnitude of these indirect associations suggests that additional organisational and individual factors are likely to be associated with these outcomes. As these mediating relationships have not previously been examined, these findings contribute to the literature on professional nursing governance. Wang et al. found that a supportive work environment is associated with higher psychological capital, which in turn is linked to effective care behaviours among hospice nurses [61]. It has been reported that nurses working with authentic leaders demonstrate higher psychological capital, which is positively associated with patient interactions and care quality [13]. Studies have also found that a positive nursing work environment is associated with nurses’ job commitment due to higher levels of psychological capital [64]. According to An et al., positive psychological capital statistically mediates the relationship between burnout and nursing performance; therefore, interventions to increase psychological capital may be associated with reduced burnout [65]. Higher levels of professional nursing governance, together with stronger psychological capital, were associated with higher perceptions of care quality and were positively linked to job satisfaction. These findings may also be interpreted within the context of Türkiye’s culture. The present findings may be considered in relation to organisational characteristics described in the Turkish healthcare literature, such as hierarchical decision‐making structures and varying levels of professional participation. Previous literature has suggested that hierarchical organisational cultures may be associated with participation in decision‐making processes; however, such cultural characteristics were not directly measured in the present study. Turkish culture, by emphasising collectivism and community‐oriented values [66], may also be associated with how professional governance is experienced in healthcare organisations. The findings suggest that higher levels of nurses’ autonomy and participation in decision‐making are positively associated with nurses’ perceived care quality and job satisfaction. Nevertheless, the moderate proportion of explained variance observed in the structural model suggests that professional nursing governance and psychological capital represent only part of a broader set of factors associated with nurses’ perceived care quality and job satisfaction. These outcomes are inherently multifactorial and may also be associated with staffing levels, workload, leadership practices, organisational culture, work environment characteristics, and individual nurse attributes. Therefore, the findings should be interpreted as highlighting important explanatory factors rather than offering a comprehensive explanation of these outcomes. Any cultural interpretation of the findings should be considered tentative and requires direct examination in future research.

An additional consideration when interpreting the findings is the potential impact of common method variance. As professional nursing governance, psychological capital, care quality, and job satisfaction were all assessed using self‐reported measures collected from the same respondents at a single time point, the observed associations may have been partially attributable to shared response tendencies, affective states, or general perceptions of the workplace. Therefore, some observed relationships may reflect not only organisational explanatory pathway but also shared perceptual or affective associations. Future studies employing longitudinal designs, objective quality indicators or multisource data would help strengthen causal inference and reduce the risk of common method bias.

## 6. Implications for Nursing Management

These research findings highlight the potential importance of integrated approaches that address both organisational structures and individual psychological resources in nursing management. The findings suggest that further development of professional nursing governance could be a valuable strategy. Management structures that do not adequately support nurses’ participation in decision‐making, address their psychological needs or provide opportunities for professional growth may be associated with lower psychological capital and less favourable nurse‐reported outcomes. Conversely, interventions focussing solely on psychological capital without considering organisational structures may not fully address the organisational factors associated with nurses’ perceived care quality and job satisfaction. The findings suggest that governance structures offering meaningful opportunities for participation in decision‐making may be more strongly associated with nurses’ perceived care quality and job satisfaction than organisational governance structures that are primarily advisory. Furthermore, governance structures may be more effective when integrated into existing administrative decision‐making processes rather than functioning separately. However, this study did not evaluate specific governance models or interventions, and these implications should, therefore, be interpreted with caution.

Nurses who participate in management may gain experience through involvement in practice‐related decisions, quality‐improvement initiatives and organisational problem‐solving processes. These experiences may be associated with higher professional confidence and psychological resilience. Managers and administrators who support professional governance can help create environments where nurses feel their expertise and professional judgement are valued. In this context, managers may play an important role in facilitating nurses’ participation in decision‐making processes and supporting the development of positive psychological resources. Therefore, rather than focussing solely on directing nurses’ activities, nursing managers may consider creating organisational conditions that facilitate nurses’ participation in decisions affecting clinical practice and the work environment. The implementation and feasibility of such approaches, however, are likely to depend on organisational resources, leadership support and local administrative contexts.

## 7. Limitations

The study used a convenience sampling strategy, as a comprehensive sampling frame of actively employed nurses in Istanbul hospitals was not available. Consequently, probability‐based sampling methods could not be implemented, which may limit the representativeness of the sample and the generalisability of the findings. Furthermore, only questionnaires with complete responses were included in the analyses, which may introduce completion bias, as nurses who completed all study instruments may differ systematically from those who provided incomplete responses.

Third, single‐item ordinal indicators were used to assess unit‐level and hospital‐level perceived nursing care quality, as well as job satisfaction, and these were treated as continuous variables within the SEM. This approach was supported by acceptable skewness and kurtosis values; still, single‐item measurement inherently limits the number of response categories available, and results involving these three outcomes should, therefore, be interpreted with a degree of caution. The structural model did not include demographic or organisational control variables such as age, years of professional experience, hospital type, shift work status or managerial position. As these factors may influence job satisfaction and nurses’ perceived care quality, their omission may affect the robustness of the estimated relationships. Future studies should examine the proposed relationships while taking these characteristics into account.

Data were collected using both online and paper‐based data collection tools; therefore, the potential influence of questionnaire administration mode cannot be excluded. Additionally, response rates could not be calculated because the number of nurses approached was unknown, limiting the assessment of nonresponse bias. Data collection took place over a four‐month period, and temporal factors such as workload fluctuations or organisational changes may have influenced participants’ perceptions. All variables were self‐reported and collected at a single time point; however, Harman’s single‐factor test indicated that common method variance was not a substantial concern. Finally, the cross‐sectional design and single‐time‐point data collection preclude causal inference. Longitudinal and experimental or intervention studies are needed to establish temporal relationships and provide stronger evidence regarding the direction of the observed associations and potential causal pathways.

Despite these limitations, the findings contribute to the existing literature on the relationships among professional nursing governance, psychological capital, nurses’ perceived care quality and job satisfaction and provide a foundation for future research.

## 8. Conclusion

The findings of this study indicate significant positive associations among professional nursing governance, psychological capital, nurses’ perceived care quality and job satisfaction among nurses working in hospitals in Istanbul, Türkiye. Higher levels of professional nursing governance and psychological capital were associated with more favourable perceptions of care quality and higher job satisfaction. However, these findings should be interpreted as associations among nurses’ perceptions rather than as evidence of improvements in objective patient or organisational outcomes. Future research could explore the relationships between the variables examined in this study using time‐lagged longitudinal designs. Further research should assess the quality of care using objective indicators, such as institutional or patient outcomes.

## Author Contributions

Conceptualisation, methodology, writing–review and editing and validation: Melek Karatuzla and Betül Sönmez; formal analysis, investigation, data curation, writing–original draft, project administration and visualisation: Melek Karatuzla; supervision: Betül Sönmez.

## Funding

This publication was supported by the Scientific Research Projects Coordination Unit of Istanbul Yeni Yuzyil University.

## Disclosure

This study is based on the first author’s doctoral thesis approved by the Istanbul University–Cerrahpaşa Graduate Education Institute. An earlier version was presented as an oral presentation (Abstract No. S‐173) at the 9th International and 20th National Nursing Congress (Ankara, Türkiye, May 14–16, 2026).

## Conflicts of Interest

The authors declare no conflicts of interest.

## Data Availability

Anonymized data supporting the findings of this study are available from the corresponding author upon reasonable request.
